# Hemodynamic and Imaging Assessment of Transcatheter Aortic Valve Replacement with the Inovare^®^ Proseal using Multislice Computed Tomography

**DOI:** 10.21470/1678-9741-2019-0103

**Published:** 2020

**Authors:** Apoana Gomes Fiori, Matheus Simonato, Alfredo Eyer, José Honório Palma da Fonseca, Diego Felipe Gaia

**Affiliations:** 1 Division of Cardiac Surgery, Escola Paulista de Medicina, Universidade Federal de São Paulo, São Paulo, Brazil.; 2 Department of Radiology, Escola Paulista de Medicina, Universidade Federal de São Paulo, São Paulo, Brazil.

**Keywords:** Transcatheter Aortic Valve Replacement, Aortic Valve, Multidetector Computed Tomography, Coronary Occlusion, Hemodynamics, Prostheses and Implants

## Abstract

**Objective:**

To evaluate the hemodynamic performance (*i.e*., gradients and paravalvular leakage [PVL]) of the new and experimental Braile Inovare^®^ Proseal. Additionally, we aimed to assess pre and postoperatively the aortic annulus and the transcatheter prosthesis using multislice computed tomography (MSCT).

**Methods:**

Patients were selected by a multidisciplinary heart team and referred for transcatheter aortic valve replacement (TAVR). MSCT was performed before and after surgery. Measurements of the aortic valve and prosthesis were conducted and correlated with the valve gradient and residual PVL.

**Results:**

Twenty-one patients were selected for the protocol. Patients had a mean age of 79 years and 38% of them were of female sex. The mean EuroSCORE II value was 12.5%±10.8. Mean gradient was reduced from 45.8±11.04 mmHg to 5.59±2.61 mmHg and there were no instances of PVL worse than mild. There were no cases of coronary obstruction or procedural death. Circularity was present in all prostheses evaluated. Circularity indexes for the prostheses were: inflow 0.05±0.03, middle third 0.04±0.02, and outflow 0.04±0.02 (*P*=0.08). The mean distance between the prosthesis and the left and right coronary ostia were 14.8 mm±3.3 and 17.3 mm±3, respectively. Oversizing was appropriate with a mean of 22.14%±6%.

**Conclusion:**

Braile Inovare^®^ Proseal transcatheter device has demonstrated low gradients with low rates of PVL. Oversizing by annular measurements was adequate. MSCT was adequate to evaluate device sizing and has demonstrated preserved expansibility and circularity in the evaluated cases.

**Table:** 

Abbreviations, acronyms & symbols	
ANOVA	= Analysis of variance
CT	= Computed tomography
EuroSCORE	= European System for Cardiac Operative Risk Evaluation
LVOT	= Left ventricular outflow tract
MCST	= Multislice computed tomography
NYHA	= New York Heart Association
PTFE	= Polytetrafluoroethylene
PVL	= Paravalvular leakage
SD	= Standard deviation
TAVR	= Transcatheter aortic valve replacement
TEE	= Transesophageal echocardiography
VARC-2	= Valve Academic Research Consortium-2

## INTRODUCTION

Transcatheter aortic valve replacement (TAVR) is an established technique supported by multiple clinical trials and an accumulated experience of over 15 years^[1,2]^. In Brazil, the Braile Inovare^®^ (Braile Biomedica, Sao Jose do Rio Preto, SP, Brazil) has been available since 2009^[3]^. Even though initial results were encouraging, the several limitations of the first-generation devices required improvement. One of the major issues was a significant incidence of paravalvular leakage (PVL), associated to a mortality increase^[4,5]^.

In the second generation of the Inovare, named Inovare Proseal, synthetic fibers (similar to those used in embolization coils) were placed circumferentially in the inferior third of the device, aiming to reduce the incidence and severity of PVL. Initial results demonstrated success in reducing this complication^[6]^.

Postoperative evaluation of a transcatheter implant is also fundamentally important, given that incompletely expanded prostheses may present with elevated gradients and leaflet geometry distortion, with an impact on long-term durability and also a plausible impact on the risk of PVL^[7]^.

Our objective in this paper was to evaluate the hemodynamic performance of the valve. Additionally, using multislice computed tomography (MSCT), we aimed to assess the dimensions of the aortic annulus of patients planned to undergo TAVR with the Inovare^®^ Proseal device, correlating these dimensions with the size of the prostheses utilized. Finally, we also aimed to determine the circularity and the degree of device expansion after the implant, associating these with the presence of residual aortic regurgitation and the transvalvular gradient.

## METHODS

Patients underwent the proposed tomographic evaluation after informed consent. The current protocol was approved by the institutional review board of our university. These patients were selected by a multidisciplinary heart team, including cardiac surgeons, cardiologists, interventional cardiologists, and anesthesiologists. Exclusion criteria were inability to undergo transesophageal echocardiography (TEE) (*e.g*., esophageal stenosis, esophageal varices), technically inadequate computed tomography (CT), and contraindicated CT (*e.g*., iodide allergy, renal failure). Other aspects considered were surgical risk, life expectancy, and quality of life. All patients underwent planned double antiplatelet therapy with acetylsalicylic acid (200 mg) and clopidogrel (75 mg) daily.

Postoperative complications were evaluated according to the Valve Academic Research Consortium-2 (VARC-2) criteria^[8]^. Procedural success was defined as a correct implant, satisfactory hemodynamics, absence of significant transvalvular or paravalvular regurgitation, and absence of major immediate complications.

MSCT was performed at least one year after the index procedure in the Department of Radiology of our institution with a 64-channel scanner (Brilliance 64, Philips, New Jersey, NJ, United States of America). Images were acquired with a collimation of 64 x 0.5 mm and a rotation time of 400 ms. Heart rate and blood pressure were monitored before the test and a beta-blocker (oral metoprolol, 50 to 100 mg) was utilized in patients without contraindications to their use and who had a heart rate > 65 beats per minute. Images were acquired in inspiratory pause and after the administration of 90 to 120 mL of iodinated contrast (350 mg/ mL) in the antecubital vein, with a speed of injection of 5 mL/s.

A long-axis view of the aortic annulus, similar to the TEE view, was obtained. Two orthogonal planes divide the long axis of the left ventricular outflow tract (LVOT) in parallel, and a third transverse plan divides the aortic annulus directly below the lowest insertion point of the three cusps to obtain a shortaxis view of the annulus. The calcium score of the valve was also measured and reported by severity, aiming to evaluate the degree of valve calcification and to determine the most compromised cusp^[9]^. An eccentricity index was utilized to determine the geometry of the annulus before the procedure using the short axis view. An eccentricity index equal to zero represents a perfect circle, whereas larger indexes represent a more ellipsoid geometry^[10]^. The index is calculated by dividing the larger diameter of the annulus by the smaller one. We have utilized Student’s t-test to compare mean and maximum gradients at different points in time: preoperative, 30-day follow-up, and 1-year follow-up. Additionally, we have used analysis of variance (ANOVA) to compare circularities at inflow, middle, and outflow.

The following measures were obtained by the MSCT in the preoperative setting: larger and smaller diameters of the aortic annulus; perimeter and area of the annulus; sinus of Valsalva; sinotubular junction; ascending aorta; height of the coronary ostia; and degree of calcification. The following measures were obtained by the MSCT in the postoperative setting: prosthesis diameter; circularity measured in the base, middle third, and distal part of the prosthesis; and distance between prosthesis and left/right coronary ostia.

Images were analysed with the OsiriX 10 (Pixmeo, Bernex, Switzerland) and the TeraRecon (TeraRecon, Foster City, CA, United States of America) softwares. Statistical analysis was performed using the IBM SPSS Statistics 22.0 (IBM, Armonk, NY, United States of America) software.

## RESULTS

Twenty-one cases were treated in a hybrid operating room. Patients had a mean age of 79 years and 38% of them were of female sex. In terms of comorbidities, over half of patients had diabetes and little over one third had pulmonary disease. The group was considered to be at high risk by the heart team, with a mean European System for Cardiac Operative Risk Evaluation (EuroSCORE) II value of 12.5%±10.8. Table 1 describes in detail the baseline characteristics of the cohort.

**Table 1:** Baseline characteristics of the patients included in the study.

Preprocedural characteristics	n = 21
Age (years, mean/range)	79.1 (63-93)
Female sex (n, %)	8 (38.1)
Diabetes (n, %)	12 (57.1)
Glomerular filtration rate < 50 mL/min (mean ± SD)	44.2±15.4
Stage V chronic kidney disease (n, %)	0 (0)
Restrictive or obstructive pulmonary disease (n, %)	7 (33.3)
Atrial fibrillation (n, %)	5 (23.8)
NYHA class (n/%)	
II	2 (9.5)
III	11 (52.4)
IV	8 (38.1)
Comorbidities	
Coronary artery disease (n, %)	8 (38.1)
Prior myocardial infarction (n, %)	5 (23.8)
Peripheral artery disease (n, %)	12 (57.1)
Prior stroke (n, %)	6 (28.6)
History of cancer (n, %)	2 (9.5)
Porcelain aorta (n, %)	1 (4.8)
EuroSCORE II (%) (mean ± SD)	12.5±10.8
Maximum gradient (mean ± SD)	74.2±23.4
Mean gradient (mean ± SD)	45.8±11
Left ventricular ejection fraction (mean ± SD)	55.2±17.5

EuroSCORE=European System for Cardiac Operative Risk Evaluation; NYHA=New York Heart Association; SD=standard deviation

Successful implant was possible in all cases (Table 2). There was no intraoperative mortality. Thirty-day mortality occurred in three cases (14.3%), with two cardiovascular deaths (9.5%). On follow-up, only one of the patients died due to a femoral fracture. The other two patients died of cardiac failure and acute myocardial infarction (previous history of coronary artery disease), respectively.

**Table 2:** Procedural characteristics of the patients included in the current analysis.

Operative characteristics	n = 21
Procedural success (n, %)	21 (100)
Conversion to open-heart surgery (n, %)	0 (0)
Need for resuscitation (n, %)	0 (0)
Need for cardiopulmonary bypass (n, %)	0 (0)
Dye volume (mL) (mean ± SD)	47.6±11.4
Time to procedural completion (min) (mean ± SD)	88.2±38.5
Postdilation (n, %)	7 (33.3)

SD=standard deviation

All patients received the Inovare^®^ Proseal, with the following prosthetic sizes utilized: one of 22 mm, six of 24 mm, six of 26 mm, seven of 28 mm, and one of 30mm. The selection of the device size took place through the measure of the aortic annulus, with oversizing based on valvular area and degree of calcification.

**Fig. 1:**
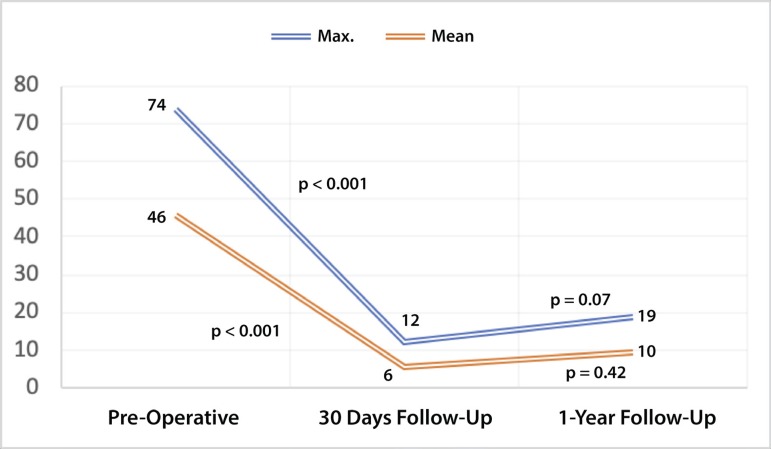
Maximum and mean gradient on 30-day and one-year follow-up. Substantial reduction in gradients is evident, with preserved results over time.

**Fig. 2:**
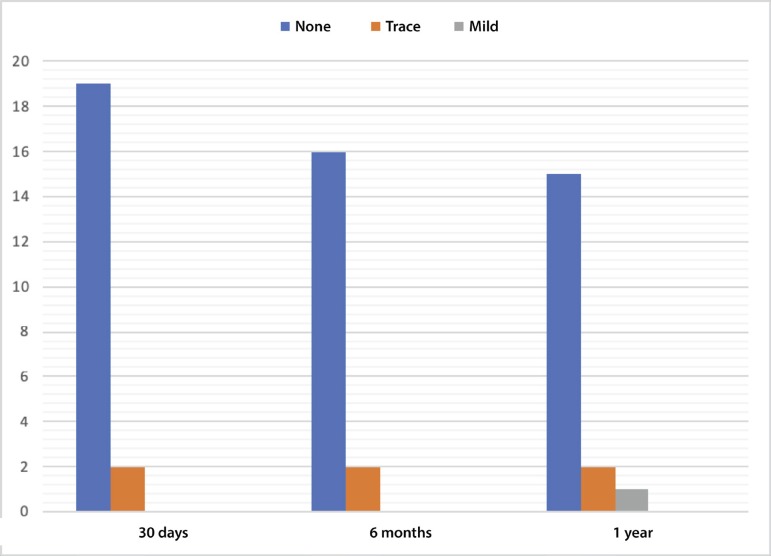
Grading of paravalvular leakage (PVL) by echocardiography. No patient presented with PVL worse than mild on one-year follow-up.

In terms of hemodynamics, there was a significant reduction in both maximum and mean gradients. Maximum gradient was decreased from 74.02±23.4 mmHg to 12.14±4.74 mmHg (*P*<0.001) and the mean gradient was also reduced from 45.8±11.04 mmHg to 5.59±2.61 mmHg (*P*<0.001). These results were preserved on follow-up (*P*=0.07 and *P*=0.42, respectively) (Figure 1). PVL was not common and there was no aortic regurgitation above mild (Figure 2).

There were no cases of third-degree atrioventricular block or pacemaker need. There were no cases of major bleeding, major stroke, above stage 2 acute kidney injury, or major vascular complications. There was no case of leaflet thrombosis or coronary obstruction.

### Imaging Analysis

Table 3 includes the CT parameters evaluated in the pre and postoperative settings. The anatomy of our patients was consistent with what is most commonly found, given that the left coronary ostium was slightly lower than the right coronary one. Additionally, the encountered parameters were within the indicated threshold, as it is recommended that the minimum distance between the annulus and the coronary ostium is at least 10 mm. The largest value for the left coronary artery was 14.3 mm and the lowest one was 9.8 mm. The largest value found for the right coronary artery was 14 mm and the lowest one was 10.5, well within the required limits.

**Table 3:** Summary of computed tomography measures included in this study.

	n=21
**Preprocedural CT parameters**	
Left coronary height (mm) (mean ± SD)	11.5±2.7
Right coronary height (mm) (mean ± SD)	12.4±2.3
Annular area (mm^2^) (mean ± SD)	483.6±71.7
Calcium score (mean ± SD)	3.3±0.6
Diameter sinus of Valsalva (mean ± SD)	37.1±2.6
Diameter sinotubular junction (mean ± SD)	32.9±3.2
Ascending aorta (mean ± SD)	35.4±3.5
**Postprocedural CT parameters**	
Circularity index, inflow (mean ± SD)	0.05±0.03
Circularity index, middle third (mean ± SD)	0.04±0.02
Circularity index, outflow (mean ± SD)	0.04±0.02
Expansion (nominal area/effective area), inflow (mean ± SD)	0.84±0.08
Expansion (nominal area/effective area), middle third (mean ± SD)	0.76±0.10
Expansion (nominal area/effective area), outflow (mean ± SD)	0.94±0,12
Distance prosthesis - left coronary artery (mean ± SD)	14.8±3.3
Distance prosthesis - right coronary artery (mean ± SD)	17.3±3

CT=computed tomography; SD=standard deviation

**Fig. 3:**
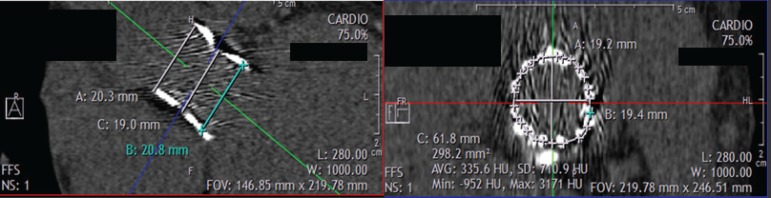
Example of documented circularity in a 20-mm Braile Inovare^®^ device with full expansion.

The circularity indexes for the prostheses were: inflow 0.05±0.03, middle third 0.04±0.02, and outflow 0.04±0.02 (*P*=0.08). Circularity, defined as up to 10% difference, was present in all prostheses evaluated (Figure 3). The mean distances between the prosthesis and the left and right coronary ostia were 11.5 mm and 12.4 mm, respectively. The expansion indexes were also recorded: inflow 0.87±0.08, middle third 0.76±0.10, and outflow 0.94±0.12. Notably, the outflow is significant more expanded compared to other segments, reflecting the absence of expansion restriction (LVOT and annulus). All devices were expanded to the full labeled size.

We also observed that the left coronary artery is on average closer to the prosthesis than the right coronary artery (Table 3). This is compatible with the anatomy, as the right coronary artery is typically in a higher position than its left counterpart. Finally, some cases presented with a distance between the artery and the prosthesis below 10 mm. However, a sufficiently wide sinus of Valsalva may allow the implant in such cases, without leading to obstruction.

In terms of sizing, the relationship between the annulus and the chosen device demonstrate that the degree of oversizing was appropriate and within the expected and recommended range for the prosthesis, with a mean of 22.14%±6%. The correct prosthesis choice is reflected in a low degree of PVL and the absence of major complications, such as the rupture of the annulus, as this complication is not only associated to the degree of calcification but also to excessive oversizing.

## DISCUSSION

TAVR has become a viable alternative to aortic valvular disease in several degrees of risk. Initial clinical trials demonstrated good results in inoperable and high surgical risk patients. Further improvements in TAVR technology and procedural technique, and an increase in operator experience, lead to the expansion of the intervention to intermediate risk patients. Ongoing randomized trials are evaluating TAVR in a low risk population, and results are expected within the next two years.

There are important considerations that will define the discussion behind the expansion of TAVR to more patients. One of them is device durability. Several factors play a role in the process of bioprosthetic leaflet degeneration, and it is possible that even the crimping of the transcatheter valve inside the delivery system may cause tissue damage associated to early dysfunction^[11]^.

The final shape of the device, that is, its circularity and completeness of expansion, may also negatively affect durability. Underexpanded devices may cause a reduction in the effective orifice area and an increase in final gradients, leading to a shift in leaflet stress that may reduce durability. Lack of circularity after delivery may lead to the same theoretical issue through an analogous mechanism. The true meaning of these issues in humans remains to be known, but in experimental animals, accelerated calcification and fibrosis have already been demonstrated^[12,13]^.

Our analyses have shown that the studied devices had good expansion and no significant reduction of their circularity, in similar fashion to the reference balloon-expandable prosthesis in the market, the Edwards SAPIEN (Edwards Lifesciences, Irvine, CA, United States of America). One study has found that the SAPIEN has a mean expansion index of 104.1%, compared to 94% (outflow) found in our study^[14]^. Another study, however, has shown an expansion index of only 84%, substantially inferior to our findings^[15]^.This variability may be due to different sizing protocols, and also due to differences in the prevalence of bicuspid valve between studies.

The degree of expansion of the Inovare^®^ was not associated to gradient elevation. However, our rate of atrioventricular block and annulus rupture in this initial series was zero. This, associated to the short profile of the device, could potentially mean that the decreased expansion would lead to a lesser degree of impingement in the conduction system, without affecting gradients significantly. Our low rate of PVL would also indicate adequate expansion, as good expansion leaves less room for residual periprosthetic gaps.

In this series, we have also observed that the middle third of the prosthesis presents with decreased expansion when compared to both inflow and outflow zones. This can be explained by the greater rigidity and calcification of the annulus, when compared to the LVOT and the sinus of Valsalva. This characteristic may be protective against annular rupture, a complication that we also did not have. On the other hand, it is possible that increased rigidity in the functional area of the device may lead to elevation in postprocedural gradients^[16]^.

The comparison in the degree of eccentricity attained in the three zones was not different, demonstrating that the lack of circularity of the annulus in relation to the LVOT and the sinus of Valsalva did not affect the prosthesis. Even in the setting of incomplete expansion, the device is capable of maintaining circularity. The same finding has been published by other authors^[14]^. The eccentricity of the device may be correlated to the degree of PVL^[15,17]^. New mechanisms of leak prevention, such as polytetrafluoroethylene (PTFE) skirts and embolization coils, may, alongside objectively reducing PVL by occupying small empty spaces of malapposition, avoid that structural deformities stemming from imperfect circularity cause such leaks. In our study, there were no leaks worse than mild.

It is also important to put our findings into perspective by comparing to self-expandable devices. One study demonstrated a lack of circularity in at least 50% of CoreValve (Medtronic, Minneapolis, MN, United States of America) cases^[18]^. However, it is still unclear of the clinical significance of this finding, as the CoreValve has similar clinical results to other devices without evidence of early degeneration^[19]^.

Finally, in this series there was no evidence of leaflet thrombosis. Theoretically, poor expansion and leaflet coaptation may be predisposing factors to thrombosis, which has been described particularly in valve-in-valve cases^[20]^.

### Limitations

Limitations in this series include the reduced number of patients. Further studies with more patients may confirm the initial results. Additionally, the length of follow-up does not allow long-term speculation on durability. Delayed recoil may take place over longer-term follow-up, although one would expect most recoil to take place just after prosthetic deployment. Additionally, delayed recoil was not evaluated in this series, as the patients did not undergo immediate CT evaluation after the procedure. Nevertheless, the fact that we have found only mild hypoexpansion with highly circular prostheses indicates that recoil is unlikely.

## CONCLUSION

The Braile Inovare Proseal^®^ transcatheter device has demonstrated good hemodynamics with low gradients and low rate of significant PVL. Expansibility was close to its nominal size when performing recommended oversizing. Additionally, MSCT evaluation was appropriate to determine sizing and has shown the adequate circularity of the device in multiple settings, leading to optimal hemodynamic performance and potentially good durability.

**Table:** 

Authors' roles & responsibilities	
AGF	Substantial contributions to the conception or design of the work; or the acquisition, analysis, or interpretation of data for the work; drafting the work or revising it critically for important intellectual content; final approval of the version to be published
MS	Substantial contributions to the conception or design of the work; or the acquisition, analysis, or interpretation of data for the work; drafting the work or revising it critically for important intellectual content; final approval of the version to be published
AE	Substantial contributions to the conception or design of the work; or the acquisition, analysis, or interpretation of data for the work; final approval of the version to be published
JHPF	Substantial contributions to the conception or design of the work; or the acquisition, analysis, or interpretation of data for the work; final approval of the version to be published
DFG	Substantial contributions to the conception or design of the work; or the acquisition, analysis, or interpretation of data for the work; final approval of the version to be published; agreement to be accountable for all aspects of the work in ensuring that questions related to the accuracy or integrity of any part of the work are appropriately investigated and resolved
